# The Acute Toxicity of Salinity in Onshore Unconventional Gas Waters to Freshwater Invertebrates in Receiving Environments: A Systematic Review

**DOI:** 10.1002/etc.5492

**Published:** 2022-11-10

**Authors:** Daniel J. Willems, Anupama Kumar, Dayanthi Nugegoda

**Affiliations:** ^1^ Ecotoxicology Research Group, School of Science RMIT University Bundoora Victoria Australia; ^2^ CSIRO Land and Water Urrbrae South Australia Australia

**Keywords:** Acute toxicity, salinity, unconventional gas, freshwater invertebrates, produced water, hydraulic fracturing

## Abstract

Industries such as unconventional natural gas have seen increased global expansion to meet the increasing energy needs of our increasing global population. Unconventional gas uses hydraulic fracturing that produces significant volumes of produced waters, which can be highly saline and pose a toxic threat to freshwater invertebrates if exposure via discharges, spills, leaks, or runoff were to occur. The primary aim of the present review was to determine the sodium (Na^+^) and chloride (Cl^−^) content of these waters as an approximate measure of salinity and how these values compare to the NaCl or synthetic marine salt acute toxicity values of freshwater invertebrate taxa. Shale gas produced waters are much more saline with 78 900 ± 10 200 NaCl mg/L and total dissolved solids (TDS) of 83 200 ± 12 200 mg/L compared to coal bed methane (CBM) produced waters with 4300 ± 1100 NaCl mg/L and TDS of 5900 ± 1300 mg/L and pose a far greater toxicity risk from NaCl to freshwater invertebrates. In addition, the toxicity of other major ions (Ca^2+^, K^+^, Mg^2+^, ${\text{CO}}_{3}^{2-}$, HCO_3_^−^, and ${\text{SO}}_{4}^{2-}$) and their influence on the toxicity of Na^+^ and Cl^−^ were evaluated. Exposure of untreated and undiluted shale gas produced waters to freshwater invertebrates is likely to result in significant or complete mortality. Shale gas produced waters have higher concentrations of various metals compared with CBM produced waters and are more acidic. We recommend future research to increase the reporting and consistency of water quality parameters, metals, and particularly organics of produced waters to provide a better baseline and help in further investigations. *Environ Toxicol Chem* 2022;41:2928–2949. © 2022 The Authors. *Environmental Toxicology and Chemistry* published by Wiley Periodicals LLC on behalf of SETAC.

## Graphical abstract

Comparing the acute toxicity (48–96‐h) of sodium chloride (NaCl) and/or synthetic marine salt laboratory exposures of freshwater invertebrates to the salinity in unconventional gas produced waters from coal bed methane and shale gas extraction, and how other major ions (Ca^2+^, K^+^, Mg^2+^, CO_3_^2–^, HCO_3_^–^, SO_4_^2–^) can influence the toxicity of Na^+^ and Cl^–^ ions and other major ions found in these waters.

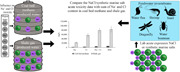

## INTRODUCTION

Salinization of freshwater environments is becoming an increasingly widespread problem because of secondary salinization caused by anthropogenic activity, in contrast to primary salinization which is derived from natural processes (Daliakopoulos et al., 2016). The major source of secondary salinization is irrigation coupled with poor drainage; other industries or processes including water‐treatment plants, mining, and extraction of natural resources such as unconventional natural gas extraction; application of fertilizers; careless disposal or reuse of saline effluents such as shale gas produced waters for road deicing; and excessive pumping of groundwaters (Daliakopoulos et al., 2016). Climate change is also resulting in indirect secondary salinization due to interruptions of the hydrological cycle and rising sea levels, resulting in expansion of salinized areas and decreased rainfall to some regions (Daliakopoulos et al., 2016).

Onshore unconventional gas exploration is a growing global industry that contributes to secondary salinization and utilizes hydraulic fracturing, which produces large volumes of flowback water and produced water, also referred to as flowback produced water (Blewett et al., 2018), or coproduced water (Independent Expert Scientific Committee on Coal Seam Gas and Large Coal Mining Development [IESC], 2014). For US‐based operations, it is estimated that between flowback and produced water 1.7 to 14.3 × 10^6^ L is produced per well (Kondash et al., 2017). Most of the produced water (92%–96%) is composed of naturally occurring brines, with 4%–8% composed of returned injected hydraulic fracturing fluids. Typically, 20%–50% of the produced water is produced in the first 6 months of hydraulic fracturing of a well (Kondash et al., 2017). Produced waters contain a large variety of chemicals (Elsner & Hoelzer, 2016; National Industrial Chemicals Notification and Assessment Scheme [NICNAS], 2017; Waxman et al., 2011). Salinity is typically the most prominent issue attributed to produced waters, particularly more so for shale gas basins than coal bed methane (CBM) basins, because the bulk of shale gas produced waters are from brine‐containing geogenic reservoirs with proportionally high concentrations of Na^+^ and Cl^−^ compared with other dissolved ions (Barbot et al., 2013; Chapman et al., 2012; Gandhi et al., 2018; Lokare et al., 2017; Rowan et al., 2015).

There are several management strategies for produced water from onshore unconventional gas extraction. Historically this involved the use of evaporation ponds, though they have become increasingly more unacceptable to use and thus less volume of produced water is managed this way (Veil, 2020). Evaporation ponds cause several major problems: atmospheric pollution due to volatile/semivolatile compounds (Field et al., 2014), attraction of animals or pest species to polluted waters (Ramirez, 2010), and terrain changes due to excavation to construct lined ponds (Davies et al., 2015). Recently, reinjection of produced water into active wells or dedicated disposal wells has become increasingly more common, with 80%–90% of produced water from unconventional oil and gas being managed between these two methods (Danforth et al., 2019; Veil, 2020). These reinjection methods of disposal also pose various risks, with groundwater and surface water contamination, induced seismic activity (Vengosh et al., 2014), and various impacts to human health if exposed to these waters (Yazdan et al., 2020). If produced water cannot be reused on site or reinjected into wells, under certain conditions and regions around the world, local regulations may allow produced water to be discharged into freshwater systems such as rivers and streams (Ferrar et al., 2013). The produced water may require pretreatment before discharge to reduce environmental impacts (IESC, 2014; Yazdan et al., 2020).

In the United States the most frequent and potentially impactful pathway for unconventional gas produced waters to enter the environment or surface waters is through discharges of these waters under the National Pollutant Discharge Elimination System (NPDES). In 2017, 1.21 × 10^8^ m^3^ (reported as 762,487,036 barrels) or 5.5% of total produced water was discharged from unconventional oil and gas extraction in the United States (Veil, 2020), with 85% by volume of discharging to surface waters occurring in Wyoming, and not all states are permitted to undertake surface discharges. Other relatively less likely and lower‐volume pathways are accidental spills (Burden et al., 2015; Folkerts et al., 2020). In Alberta, Canada, there was an estimated >2500 produced water spills that occurred from 2005 to 2012, of which more than 113 had entered freshwater lakes or streams (Alessi et al., 2017; Folkerts et al., 2020; Goss et al., 2015). Data from 2005 to 2014 from >30 000 wells across several US states showed that 50% of spills were associated with storage or transport of produced water (Patterson et al., 2017), with an estimate of 2%–16% of wells reporting a spill every year; the largest by volume spill reported was 3756 m^3^ (Patterson et al., 2017). In the United States, another common pathway by which produced water enters the environment is from road runoff because produced waters are currently used in 13 states for road maintenance, dust suppression, or deicing (Tasker et al., 2018). The produced water used for these applications can be mobilized during rain or thaw events into surrounding groundwater and surface water, resulting in potential toxicity to aquatic biota.

To reduce the salinity of produced water and make it safer for discharge, disposal, or repurposing of these waters, membrane‐based separation techniques such as reverse osmosis can be used. Other membrane‐based techniques such as micro‐, ultra‐, and nanofiltration are also used to specifically target other components in the water, as detailed in Fakhru'l‐Razi et al. (2009). Reverse‐osmosis membranes are designed to reject all dissolved and ionic species including Na^+^ and Cl^−^ (Fakhru'l‐Razi et al., 2009). These technologies, despite becoming more affordable, are still expensive to set up and operate, particularly in remote regions. These treatments also result in producing highly concentrated sludges that require further disposal or management.

Salinity toxicity in the context of the present review is how toxic Na^+^ and Cl^−^ ions are to freshwater invertebrates and how other major ions in these waters may influence the toxicity of Na^+^ and Cl^−^ ions. The ions Cl^−^ followed by Na^+^ are usually the most abundant in onshore unconventional gas produced waters (particularly so for shale gas produced water) and are usually the most dominant in most naturally occurring bodies of water. Despite Na^+^ and Cl^−^ being essential for life on earth, like all chemicals they can become toxic to biota when concentrations exceed physiological needs that the organism has evolved and adapted to. Many laboratory studies have investigated NaCl or synthetic marine salts (SMS) toxicity to freshwater invertebrates (Dunlop et al., 2008; Horrigan et al., 2007; Kefford et al., 2006, 2012; Piscart et al., 2011). The toxicity of the two ions Na^+^ and Cl^−^ is well understood for freshwater organisms which are hyperosmotic relative to their aquatic environment. An excess of Cl^−^ in the surrounding aquatic environment overwhelms active or bicarbonate exchange pumps, interfering with osmoregulation and thus causing toxicity (Elphick et al., 2011). In animal cells, Na^+^ is the major cation in extracellular space and in excess can interrupt normal functioning of the Na^+^/K^+^‐adenosine triphosphatase pump, which becomes detrimental to cellular health (Pirahanchi et al., 2021). Also, Na^+^ is known to be involved in the regulation of blood pressure and volume, pH, and osmotic equilibrium.

A systematic search of the scientific literature was conducted to determine Na^+^ and Cl^−^ concentrations in wastewaters from onshore unconventional gas activities; how the concentrations of these two ions may impact freshwater invertebrates if exposed to these waters through discharges, spills, leaks, or runoff from road maintenance; and how other major ions (Ca^2+^, K^+^, Mg^2+^, ${\text{CO}}_{3}^{2-}$, HCO_3_^−^, and ${\text{SO}}_{4}^{2-}$) influence the toxicity of the Na^+^ and Cl^−^ ions. This resulted in a second systematic search of the literature to compile and determine the sensitivity of freshwater invertebrate taxa to NaCl or an SMS from laboratory‐based experiments. To guide our review, the following questions are addressed: 1) What concentrations of Na^+^ and Cl^−^ and other major ions (Ca^2+^, K^+^, Mg^2+^, ${\text{CO}}_{3}^{2-}$, HCO_3_^−^, and ${\text{SO}}_{4}^{2-}$) are in onshore unconventional gas waters, and are there any geographical or basin type (CBM or shale gas) differences? 2) What is the toxicity to freshwater invertebrates of other major ions (Ca^2+^, K^+^, Mg^2+^, ${\text{CO}}_{3}^{2}$, HCO_3_^−^, and ${\text{SO}}_{4}^{2-}$) in terms of how they influence the toxicity of Na^+^ and Cl^−^ and among each other? 3) What is the acute toxicity of NaCl and/or SMS to freshwater invertebrates? Which taxa (order and family) are most sensitive and tolerant? 4) Using the sum of Na^+^ and Cl^−^ concentrations in unconventional gas waters as an estimate, what are the concentrations of total Na^+^ and Cl^−^ content in these waters, and how do they relate to freshwater invertebrate acute toxicity endpoints for NaCl and/or SMS? What taxa will be at risk, and does the Na^+^ and Cl^−^ content of shale gas produced waters pose more risk compared with CBM produced waters? 5) Are there any geographical or basin type (CBM or shale gas) differences relating to dissolved ions or water quality parameters (WQPs) in onshore unconventional gas waters?

## MATERIALS AND METHODS

### Literature acquisition and screening

Two sets of literature searches using the Preferred Reporting Items for Systematic Reviews and Meta‐Analyses (PRISMA) guidelines (McInnes et al., 2018) were followed to search the two journal article databases Scopus and Web of Science to comprehensively identify published literature from all years up to and including the end of May 2020: Set 1 pertaining to Na^+^ and Cl^−^ concentrations in onshore unconventional gas waters and Set 2 pertaining to NaCl and/or SMS toxicity to freshwater invertebrates from laboratory exposures. Search terms were built from prior literature searches compiling common keywords found in the title and in the keywords section of articles.

For Set 1, the Scopus and Web of Science journal article databases were used to search for articles relating to salinity in onshore unconventional gas waters. The searches were made based on the terms and logical operators described in Table 1 that were found within the topic for Web of Science (all databases), which includes title, abstract, author keyword, and keyword plus. In Scopus, article title, abstract, and keywords were used. To refine the searches, English and article results were selected in each of the database's searches. The searches in Scopus and Web of Science were made on May 26, 2020. Results were exported as BibTeX files, and most of the articles were acquired using Mendeley Desktop (Ver 1.19.4 for Mac OS); the articles that could not be obtained using Mendeley were then sourced individually utilizing the RMIT University library access to journals.

**Table 1:** Search terms used in the journal database searches with context to salinity in unconventional gas waters

	Field 1	Field 2	Field 3
CBM	Hydrofracking	Brackish	Associated water
Coal bed methane	Natural gas	Brine	Co–produced water
Coal seam gas	Shale gas	Conductivity	Flowback water
CSG	Tight gas	NaCl	Produced water
Fracking	Unconventional gas	Saline	Wastewater
Fracking	Unconventional natural gas	Salinity	–
Hydraulic fracturing	–	Salt	–
	–	Sodium chloride	–

Field 1: unconventional gas terms. Field 2: salinity terms. Field 3: water terms in context of unconventional natural gas. The logical operator “OR” was used between search terms within a field, while “AND” was used between fields.

For Set 2, Scopus and Web of Science were used to search for articles relating to NaCl and/or SMS toxicity to freshwater invertebrates. The searches were made based on the terms and logical operators described in Table 2 and found in title, abstracts, and keyword fields, as specified for Set 1; and the results were limited to English and article‐only results. Both databases (Web of Science and Scopus) were searched on May 28, 2020. Results from each database were exported as BibTeX and from there followed the same procedure as for Set 1. In some instances, across both sets of searches some articles were unobtainable with the university's library access.

**Table 2:** Search terms used in the journal database searches with context of salinity (NaCl) or synthetic marine salt toxicity to freshwater invertebrates

	Field 1	Field 2	Field 3
Brackish	Acute	Stressor	Freshwater invertebrate
Brine	Chronic	Tolerance	Macroinvertebrate
Conductivity	Concentration	Tolerant	–
NaCl	EC	Toxic	–
Saline	Effective	Toxicant	–
Salinity	Fatal	Toxicity	–
Salt	Mortality	–	–
Sodium chloride	–	–	–

Field 1: salinity terms. Field 2: toxicity terms (ecotoxicity relevance). Field 3: freshwater invertebrate terms. The logical operator “OR” was used between search terms within a field, while “AND” was used between fields.

EC = electrical conductivity.

**Figure 1:**
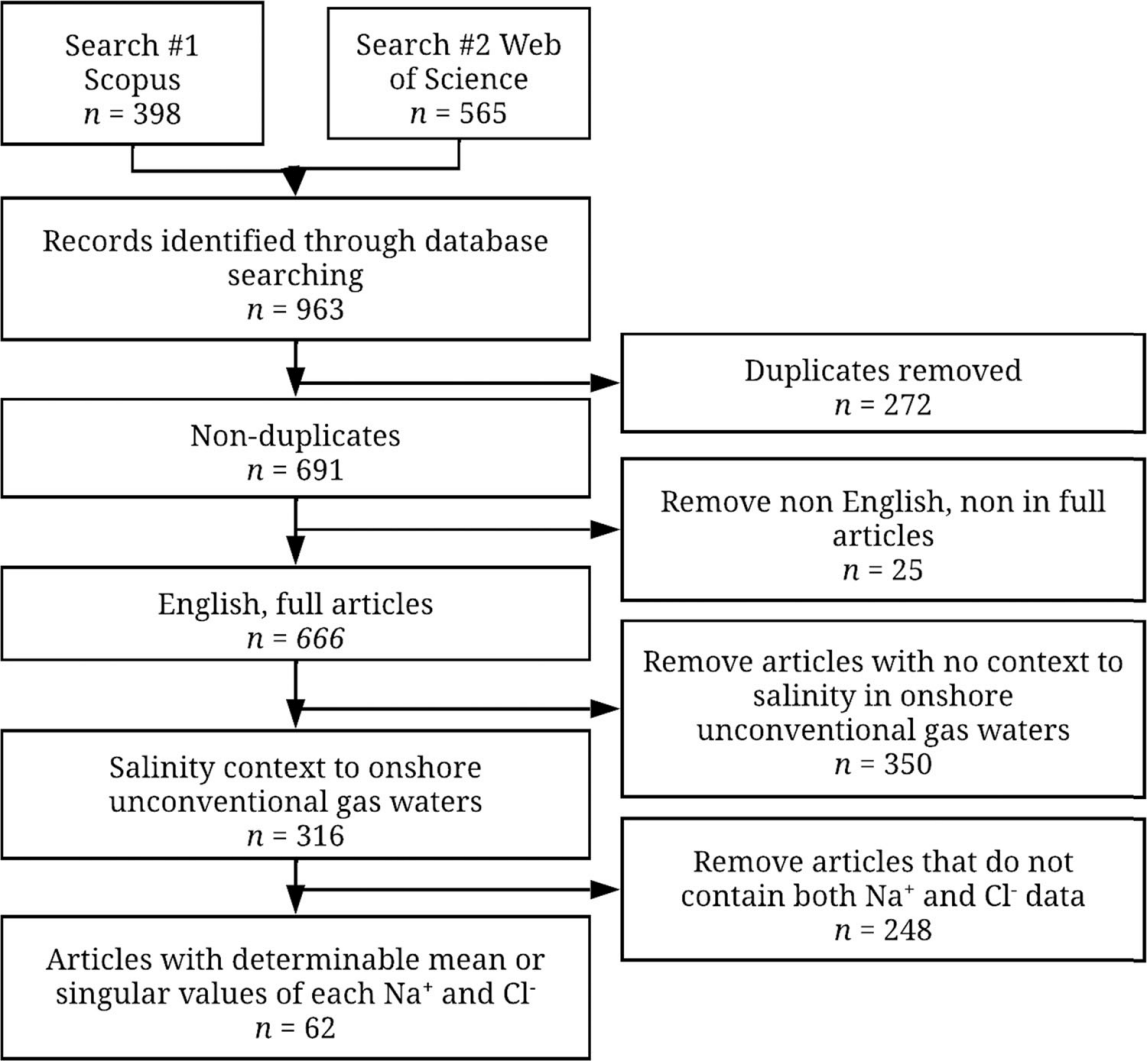
Inclusion flowchart to screen literature containing salinity (Na^+^ and Cl^−^) concentrations in untreated onshore unconventional gas waters which formed Supporting Information, Table S1. Search 1 and 2 *n* values refer to the number of results that were obtainable/downloadable through Mendeley Desktop and the institutional library access. Flow diagrams were constructed following PRISMA systematic review guidelines (McInnes et al., 2018).

**Figure 2:**
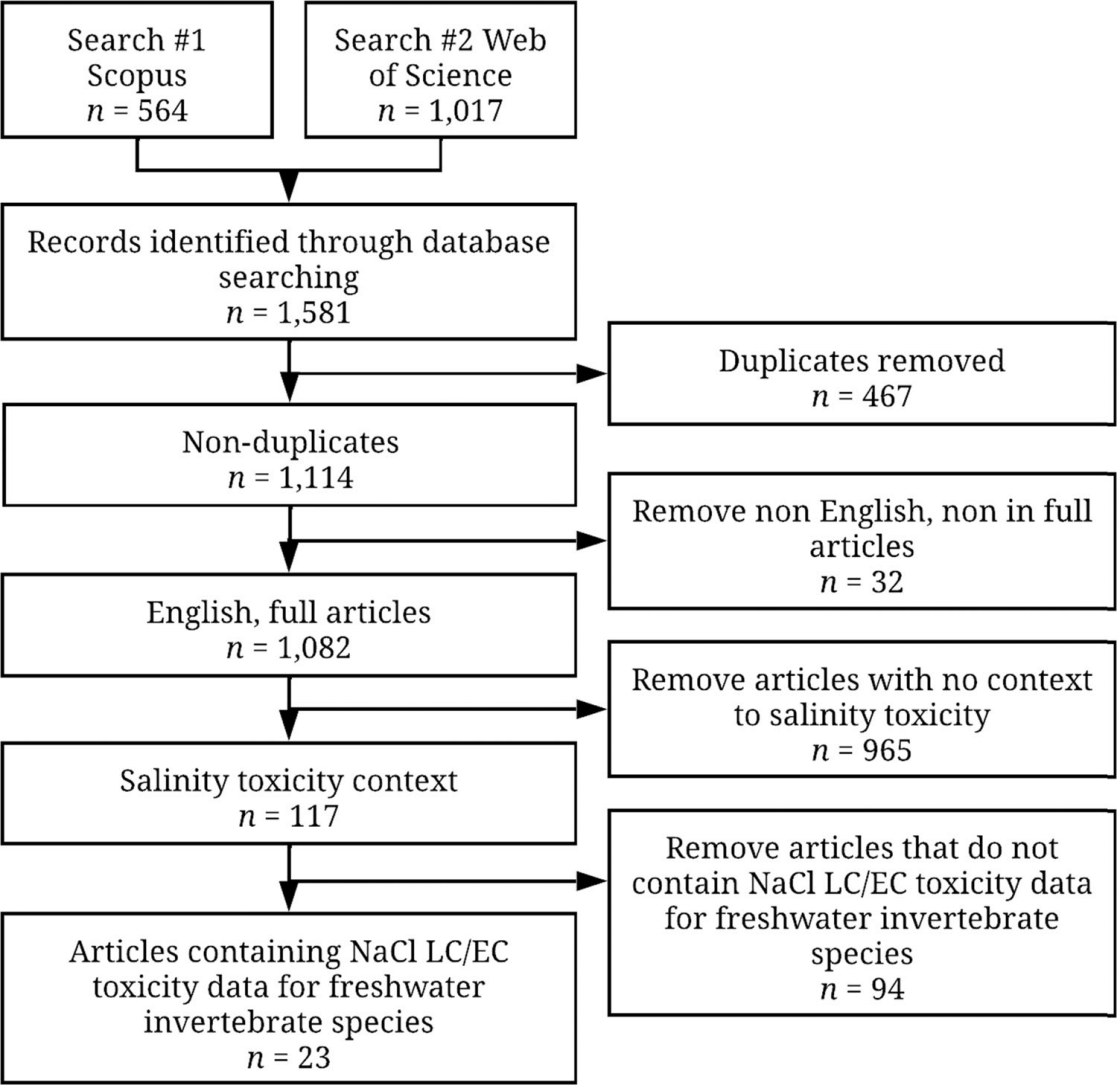
Inclusion flowchart to screen literature containing salinity (NaCl) and synthetic marine salt toxicity data for freshwater invertebrates which formed Supporting Information, Tables S2 and then S2.1. Search 1 and 2 *n* values refer to the number of results that were obtainable/downloadable through Mendeley Desktop and the institutional library access. Flow diagrams were constructed following PRISMA systematic review guidelines (McInnes et al., 2018).

Across both sets of searches (Figures 1 and 2) after all articles were acquired, duplicate file removal was performed using Duplicate File Finder Remover for Mac OS, with further duplicate removal checks using the Mendeley Desktop duplicate finder function. For both sets of searches, despite limiting searches to English and article‐only documents in Scopus and Web of Science, files that were not English and not complete articles (abstracts, reviews, reports, etc.) still appeared and were removed from further screening steps. This ensured that both sets of searches had only full articles in English. Further screening steps are addressed by Set 1 and Set 2 separately.

Set 1 screening of articles (Figure 1) was further refined by removing articles that lacked context to salinity in onshore unconventional gas water; it was important that the context was for onshore unconventional gas, given the focus of our review on freshwater invertebrates and the toxicity of salinity, which would not be possible in an offshore setting (because only marine invertebrates would be present). This still left a portion of unwanted literature that, despite being in context to salinity and onshore unconventional gas waters, was typically relevant to mostly water treatment or repurposing these waters for secondary uses; ultimately the articles that were of most relevance were those that contained measured Na^+^ and Cl^−^ concentrations in untreated onshore unconventional gas wastewaters from wells (not simulated waters, brines from reverse osmosis processes, etc.). There were some studies where mean values could not be calculated because of insufficient or not applicable information (i.e., only reporting minimum, maximum, and median values); these studies were omitted. This resulted in a final 62 articles (Figure 1) that were then used to create Supporting Information, Tables S1 and S1.1, which quantify the WQPs, and Supporting Information, Table S1.2, which quantifies element and major polyatomic ion concentrations.

Set 2 screening of articles (Figure 2) was further refined by removing articles that lacked context to NaCl or SMS toxicity. The articles that remained were checked to ensure that they contained acute or chronic lethal concentration values (LC*x*) or effective concentration (EC*x*) values and were kept, providing the values pertained to invertebrate species. Invertebrates that had a stage of their life cycle in freshwater were included even if the invertebrate becomes a terrestrial organism in its adult stage. This resulted in 23 usable original articles that were then used to create Supporting Information, Table S2, which was then further refined to create Supporting Information, Table S2.1, which was targeted toward the acute toxicity data, as further described in the section *Data collection and definitions*.

### Data collection and definitions

Various types of information and data were recorded for meta‐analysis to create Supporting Information, Tables S1, S1.1, S1.2, S2, and S2.1. For the sake of clarity, each of these supporting tables will be addressed and described separately.

In Supporting Information, Table S1, the information recorded included country (the country where the basin is located), location (i.e., the state/region/province/basin), and type of gas reservoir (CBM, shale gas, or tight gas). Class of wastewater(s) was either flowback water or produced water; the limited literature that referred to their waters as coproduced water was simplified to produced water for the present review. Concentrations of Na^+^ and Cl^−^ (milligrams per liter) were recorded as minimum, maximum, and mean. Mean concentrations of Ba^2+^ and o‐cresol were determined if present (this was of interest to another aspect of research outside the context of the present review). Measured WQPs, elements (anions and cations), and organics that were studied were also recorded qualitatively. Quantitative recordings for WQPs (Supporting Information, Table S1.1) and for the elements and polyatomic anions studied (Supporting Information, Table S1.2) are detailed below.

In Supporting Information, Table S1.1 the WQPs were quantitatively assessed by recording or determining mean values of the various parameters found in each entry/study and, if required, were converted to standardized units. The parameters included alkalinity (milligrams per liter), biochemical oxygen demand (milligrams per liter), chemical oxygen demand (milligrams per liter), dissolved oxygen (milligrams per liter), dissolved organic carbon (milligrams per liter), electrical conductivity (microsiemens per centimeter), oil and grease (milligrams per liter), oxidation‐reduction potential (millivolts), pH, sodium adsorption ratio (milliequivalents per liter), total dissolved solids (TDS, milligrams per liter), total organic carbon (milligrams per liter), total hardness as CaCO_3_ (milligrams per liter), total suspended solids (TSS, milligrams per liter), and turbidity (nephelometric turbidity units). Not all WQPs that the authors came across were recorded quantitatively from the literature; there were some that related to water color or radiation and major nutrient compounds such as nitrates.

In Supporting Information, Table S1.2 elements are organized by increasing atomic number; then, the polyatomic ions (${\text{CO}}_{3}^{2}$, HCO_3_–, and ${\text{SO}}_{4}^{2-}$), mean values (milligrams per liter), were recorded or determined from the literature. In rare instances where studies reported below detectable limits for elements, they were omitted from Supporting Information, Table S1.2, though they are listed in Supporting Information, Table S1 because our study attempted to analyze the element. This was done to ensure that only numerical, finite data points were present in Supporting Information, Table S1.2 for data analysis.

In Supporting Information, Table S2 the information recorded included country (based on the organisms' origin) because in some studies organisms were imported for laboratory research as well as taxonomic classification of order, family, genus, and species; common name; life stage; salt source (SMS or NaCl); toxicity parameter(s); the toxicity values of NaCl or SMS in microsiemens per centimeter and milligrams per liter (most toxicity data were originally reported as microsiemens per centimeter). Conversion between milligrams per liter and microsiemens per centimeter was done per Hart et al. (1991). Other toxicants, conditions, and time points studied were also recorded. The authors recorded 96‐h toxicity data preferentially for acute toxicity values if multiple other time points such as 48 or 72 h were also available. The NaCl LC50 or EC50 values were preferred over other toxicity endpoints such as LC/EC5,10,20, though many of the studies used SMS (the major ions are Na^+^ and Cl^−^). For some of the literature where toxicity data were estimated based on limited individuals because of scarcity of an organism in the literature, a threshold of *n* > 10 individuals was selected. The authors in many instances were able to acquire order and family classifications if only genus and species was provided by using reliable and up‐to‐date online databases: the Integrated Taxonomic Information System (2013) and the Murray‐Darling Freshwater Research Centre (MDFRC; 2021), with MDFRC (2021) being particularly useful for Australian invertebrate classifications. Taxonomic classifications were initially recorded in August 2020, though they required updating as of July 2021 against the same databases because the authors realized that a piece of the literature had incorrectly reported the taxonomic classification of class under a column titled *order*, fundamentally affecting subsequent data analysis (which was rerun/recorrected). In a few instances superorder and subclass were used in the order column because it was the lowest level of taxonomic classification that was reported for that specific publication.

Supporting Information, Table S2 was further refined for acute toxicity data to create Supporting Information, Table S2.1. Some of the entries from the literature reported LC/EC50 as less than or greater than values; these entries were omitted. Although some entries were of a relatively narrow range, the midpoint of the ranges was used to form a representative singular value. At this stage acute toxicity values as microsiemens per centimeter were converted to milligrams per liter (Hart et al., 1991) for use in statistical/data analysis so that they could be compared with Na^+^ and Cl^−^ (milligrams per liter) values in onshore unconventional gas waters. Chronic toxicity data were poorly represented from the screened literature and thus omitted from further investigation.

### Statistical analysis

A descriptive statistical approach was used to identify any patterns that emerged from the data. Analyses of variance (ANOVAs) were used to assess for potential significant differences for Na^+^ and Cl^−^ and other major ions by country and basin type in onshore unconventional gas waters (Supporting Information, Table S5); this was repeated for the 10 most commonly investigated elements and WQPs. Tukey's post hoc test was used when multiple groups required comparison. The acute toxicity of NaCl and/or SMS by order and family was assessed descriptively, given that many taxa had low representation across the compiled data. Statistical significance was deemed at α ≤ 0.05. All analyses were conducted using IBM SPSS Statistics Ver 24 for Mac OS (IBM, 2016).

## RESULTS

**Figure 3:**
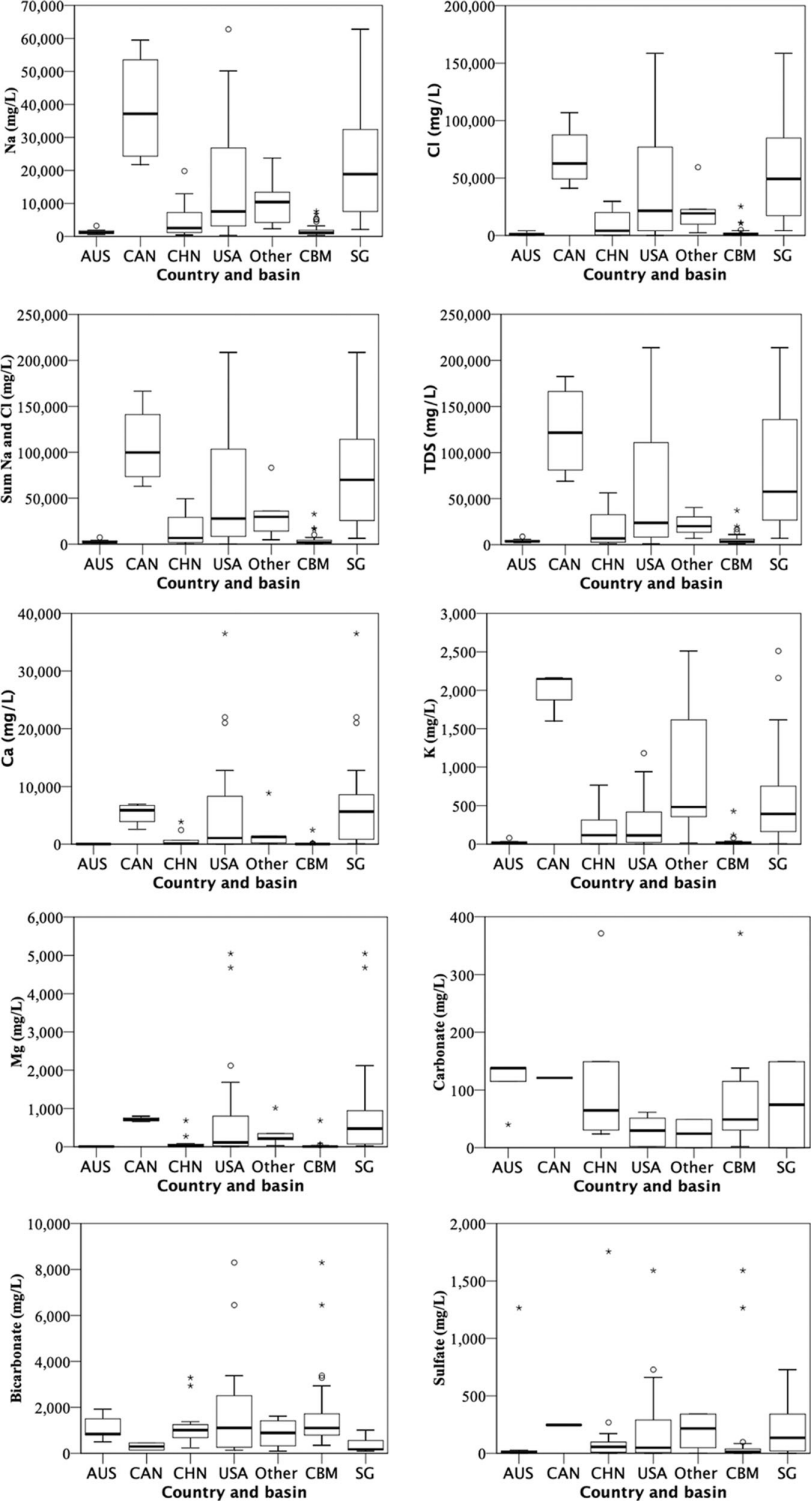
Summary of major cations (Ca, K, Mg, Na), anions (Cl, ${\text{CO}}_{3}^{2}$, HCO_3_, ${\text{SO}}_{4}^{2}$) total dissolved solids and sum of Na and Cl salinity in onshore unconventional gas waters by country (and basin coal bed methane and shale gas. Based on data compiled from Supporting Information, Tables S1.1 and S1.2. A statistical summary of Figure 3 can be found in Supporting Information, Table S5. AUS = Australia; CAN = Canada; CHN = China; USA = United States of America; CBM = coal bed methane; SG = shale gas; TDS = total dissolved solids.

**Figure 4:**
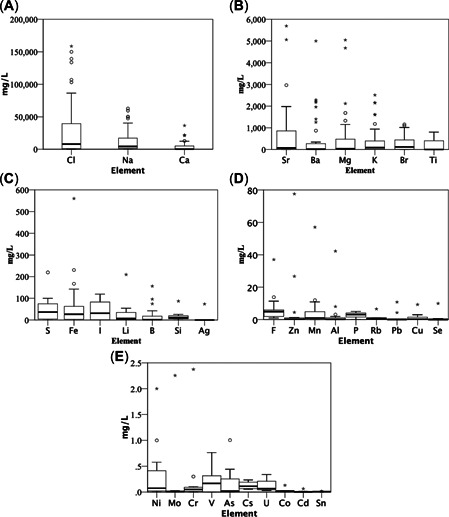
Concentrations of elements in untreated onshore unconventional gas waters from Supporting Information, Table S1.2. Only elements that had *n* ≥ 4 entries were used. (**A–E**) Parts are organized by highest (left) to lowest (right) mean concentrations of each element and are tiered together. (**A**) ≥1000 mg/L, (**B**) ≥100 mg/L and <1000 mg/L, (**C**) ≥10 mg/L and <100 mg/L, (**D**) ≥1 mg/L and <10 mg/L, (**E**) <1 mg/L. Outliers were kept, highlighting the large variability in elemental concentrations in onshore unconventional gas waters.

A total of 62 publications were published and obtainable up to the end of May 2020 reporting concentrations of both Na^+^ and Cl^−^ ions in waters associated with onshore unconventional gas exploration (Supporting Information, Table S1; see inclusion flowchart Figure 1 for further detail). This resulted in 72 entries (sets of data) because some publications included multiple gas basins, and it was deemed necessary to treat the associated values separately and not to calculate an average across basins. Across the 72 entries, nine countries were represented, the United States (USA; *n* = 32), Australia (AUS; *n* = 16), China (CHN; *n* = 15), Canada (CAN; *n* = 4), and Germany, India, Iran, Japan, and Saudi Arabia (each *n* = 1), which formed the “other” category in Figure 3 and Supporting Information, Tables S5 and S5.1. All 16 entries for AUS were CBM; all four entries for CAN were shale gas. The three types of onshore gas reservoirs were represented as follows: CBM (*n* = 35), shale gas (*n* = 33), tight gas (*n* = 1); *n* = 4 studies did not specify (N.S.) gas reservoir. Onshore unconventional gas wastewater classes studied were flowback water (*n* = 16), produced water (*n* = 62), and N.S. (*n* = 1). The sum for these sets does not always equal 72 given that some publications studied multiple categories. Subsequent data analysis in relation to Supporting Information, Tables S1, S1.1, and S1.2 involved all 72 sets of data. (Figure 4).

**Figure 5:**
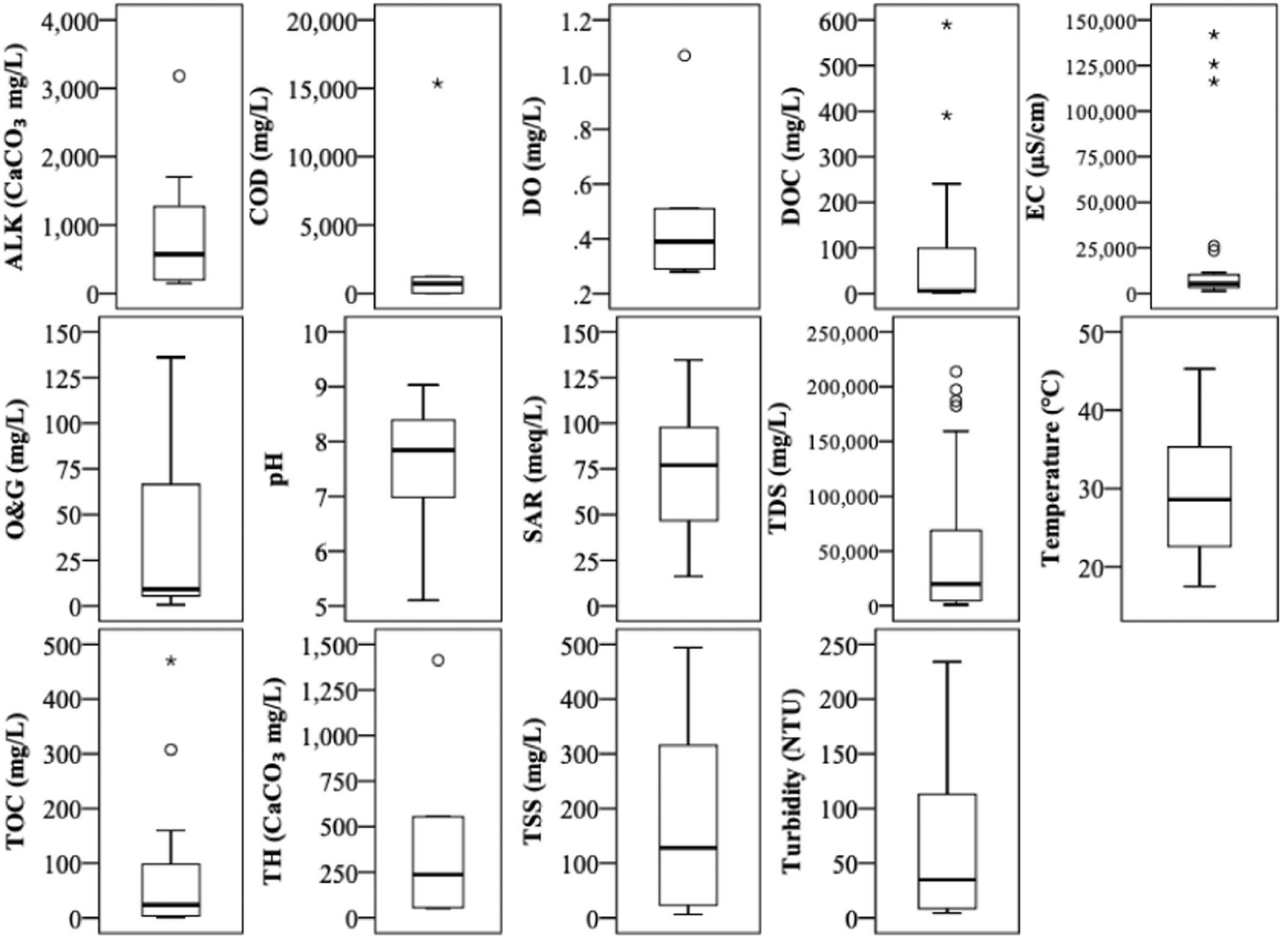
Water quality parameters from onshore unconventional gas waters, based on the data compiled in Supporting Information, Table S1.1. Biochemical oxygen demand and oxidation‐reduction potential data have been omitted because *n* = 1. ALK = alkalinity; COD = chemical oxygen demand; DO = dissolved oxygen; DOC = dissolved organic carbon; EC = electrical conductivity; O&G = oil and grease; SAR = sodium adsorption ratio; TDS = total dissolved solids; TOC = total organic carbon; TH = total hardness; TSS = total suspended solids; NTU = nephelometric turbidity unit.

From the 72 sets of WQP data and the 16 different WQPs (Supporting Information, Table S1.1) the three most studied were TDS (*n* = 52), pH (*n* = 47), and electrical conductivity (*n* = 27), as statistically summarized in Supporting Information, Table S4 and presented in Figure 5. Forty‐one of the 72 articles/entries evaluated three or fewer WQPs. Given the sparsity of WQP data, subsequent data analysis with enough power or reliability was limited to TDS/electrical conductivity and pH (see section *Water quality of onshore unconventional gas waters*).

**Figure 6:**
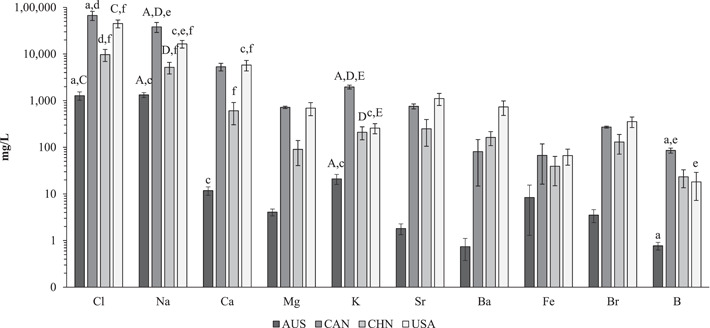
Log10 plot of mean (±SE) concentrations of the 10 most studied elements from Supporting Information, Table S1.2 by country: Australia (AUS), Canada (CAN), China, (CHN) and the United States (USA). Significant differences (α > 0.001 ≤ 0.05) between country unique combinations (*n* = 6) are as follows: a = AUS–CAN, b = AUS–CHN, c = AUS–USA, d = CAN–CHN, e = CAN–USA, f = CHN–USA. Capital letters (A–F) represent highly significant differences between pairs (*α* ≤ 0.001).

**Figure 7:**
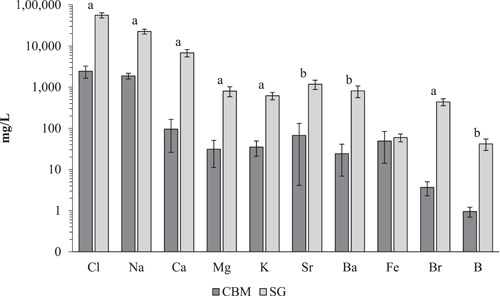
Log10 plot of mean (±SE) concentrations of the 10 most studied elements from Supporting Information, Table S1.2 by basin (coal bed methane [CBM] or shale gas [SG]). Highly significant differences between CBM and SG for each of the elements is denoted by a = significance (*α* ≤ 0.001), b = significance (*α* > 0.001 ≤ 0.05); no label represents no significance.

Sixty‐one different elements had at least one reported value (Supporting Information, Table S1.2). A statistical summary of the elements that appeared four or more times is shown in Supporting Information, Table S3. Excluding the elements Na and Cl (*n* = 72 for each), the next eight most frequently reported elements across all 72 entries were Ca (*n* = 71), Mg (*n* = 69), K (*n* = 62), Sr (*n* = 46), Ba (*n* = 42), Fe (*n* = 39), Br (*n* = 32), and B (*n* = 29). The most abundant elements by average mass were Cl > Na > Ca > Sr > Ba > Mg > K > Br (Supporting Information, Table S3). The 10 most frequently reported elements were statistically assessed for differences by geographically distinct countries (AUS, CAN, CHN, USA Figure 6) or basin (coal bed methane or shale gas Figure 7).

A total of 23 publications (resulting in 244 entries) were published and obtainable up to the end of May 2020 that reported one or more acute or chronic toxicity values for NaCl and/or SMS (Supporting Information, Table S2) and were then further refined for only acute toxicity because of poor chronic toxicity data representation. The refined acute‐only data formed Supporting Information, Table S2.1 and are summarized as follows. There was a total of 22 publications with 220 unique entries. Organisms either originated or were studied from 12 countries or distinct geographical regions of the world as reported from the literature: Australia (*n* = 144), France (*n* = 22), the United States (*n* = 22), Israel (*n* = 9), South Africa (*n* = 9), Portugal (*n* = 5), North America (*n* = 3), Ponto‐Caspian (*n* = 2), and (*n* = 1) for each of Mediterranean basin, New Zealand, northern Europe, and southeastern Europe. The number of up‐to‐date unique taxonomic classifications included order (*n* = 30), family (*n* = 61), genus (*n* = 85), and species (*n* = 83). There were many instances where identification was limited to family classification; thus, there was a high count of not applicable (N.A.) entries at the genus and species levels. The N.A. entries by taxonomic classification included order (*n* = 0), family (*n* = 5), genus (*n* = 64), and species (*n* = 91). The frequency of acute toxicity endpoints was represented as follows: 72‐h LC50 (*n* = 163), 96‐h LC50 (*n* = 26), 48‐h LC50 (*n* = 19), 48‐h EC50 (*n* = 5), 96‐h EC50 (*n* = 3), 24‐h LC50 (*n* = 2), and 24‐h EC50 (*n* = 2). Most of the 24‐ and 48‐h acute toxicity data relate to the taxonomic families of Unionidae (freshwater mussels) and Daphniidae (water fleas).

### Na^+^, Cl^−^, and other major ion concentrations in onshore unconventional gas waters

The concentrations of Na^+^ and Cl^−^, other major ions (Ca^2+^, K^+^, Mg^2+^, ${\text{CO}}_{3}^{2-}$, HCO_3_^−^, and ${\text{SO}}_{4}^{2-}$), and TDS are presented by country and by basin in Figure 3, with a summary of descriptive statistics in Supporting Information, Table S5 and associated one‐way ANOVA summary data in Supporting Information, Table S5.1. Investigation of tight gas was omitted, and differences between flow backwater and produced water were also not investigated because of insufficient representation of flowback water‐only studies.

Significant differences by geographically distinct countries (AUS, CAN, CHN, USA) in Na^+^ and Cl^−^ (Figure 6) are summarized as follows. For Na^+^ across the six unique pairs of countries, AUS–CHN were not significantly different between groups (*α* > 0.05). The other five unique pairs were either highly significantly (*α* ≤ 0.001) or significantly different from each other (*α* > 0.001 ≤ 0.05, Figure 6). For all unique pairs with Cl^−^, AUS–CHN and CAN–USA were not significantly different (*α* > 0.05) from each other, whereas the other four unique combinations were all significantly different (*α* > 0.001 ≤ 0.05, Figure 6; Supporting Information, Table S5.1) from one another. Between CBM and shale gas basins for both Na^+^ and Cl^−^ there were highly significant differences (*α* ≤ 0.001, Figure 7).

The other major cation (Ca^2+^, Mg^2+^, and K^+^) concentrations by country (Figure 6) showed some significant differences for Ca^2+^ between AUS–USA and CHN–USA, no significant differences between unique country pairs for Mg^2+^, whereas for K^+^ there were three highly significant (*α* ≤ 0.001) different unique pairings between AUS–CAN, CAN–CHN, and CAN–USA and one significantly different pairing of AUS–USA (*α* > 0.001 ≤ 0.05). Between CBM and shale gas basins (Figure 7), Ca^2+^, Mg^2+^, and K^+^ were highly significant different (Supporting Information, Tables S5 and S5.1).

For the three polyatomic anions (${\text{CO}}_{3}^{2-}$, HCO_3_^−^, and ${\text{SO}}_{4}^{2-}$) the only significant difference (*α* ≤ 0.05) to be determined was HCO_3_^−^ concentrations between the higher concentrations found in CBM basins and the lower in shale gas basins (Supporting Information, Tables S1, S5.1). Between these two basin types there were no significant differences for ${\text{CO}}_{3}^{2-}$ and ${\text{SO}}_{4}^{2-}$. For ${\text{CO}}_{3}^{2-}$ and ${\text{SO}}_{4}^{2-}$ post hoc testing could not be performed between countries because of at least one group having fewer than two cases.

### Acute toxicity of NaCl and/or SMS to freshwater invertebrates

The mean acute toxicity of NaCl and/or SMS to freshwater invertebrates across 220 entries was 13 500 ± 550 mg/L (Supporting Information, Table S2.1). A minimum of 560 mg/L was reported for *Lampsilis siliquoidea* (at a glochidia life stage) as a 48‐h EC50, and a maximum of 35 360 mg/L was reported for adult *Austrochiltonia* sp. as a 72‐h LC50. By order classifications (Supporting Information, Table S6) the mean acute toxicity ranged from 2800 mg/L (Unionida) to 27 300 mg/L (Zygoptera), whereas for family classifications (Supporting Information, Table S7) it ranged from 2800 mg/L (Unionidae) to 34 300 mg/L (Chiltoniidae).

From the orders (Supporting Information, Table S6; Figure 9) and families (Supporting Information, Table S7; Figure 7) that were represented *n* ≥ 5, the three most tolerant orders to NaCl and/or SMS were Decapoda (24 400 ± 1100 mg/L, *n* = 19), Odonata (22 800 ± 1700 mg/L, *n* = 15), and Coleoptera (22 200 ± 1600 mg/L, *n* = 14). The three most sensitive orders were Unionida (2800 ± 340 mg/L, *n* = 16), Diplostraca (5100 ± 770 mg/L, *n* = 7), and Ephemeroptera (6900 ± 550 mg/L, *n* = 26). The three most tolerant families to NaCl and/or SMS were Coenagrionidae (27 900 ± 1500 mg/L, *n* = 7), Dytiscidae (25 500 ± 1400 mg/L, *n* = 9), and Atyidae (22 800 ± 1100 mg/L, *n* = 13). The three most sensitive families were Unionidae (2800 ± 340 mg/L, *n* = 16), Daphniidae (5000 ± 890 mg/L, *n* = 6), and Planorbidae (5500 ± 1300 mg/L, *n* = 6). Sixteen of the 30 orders and 41 of the 61 families were represented (*n* ≤ 2). Possible differences in salinity sensitivity for freshwater invertebrates by country/region was not assessed because of low representation in most countries/regions and because 16 of the 22 US‐related entries were from the most sensitive family (Unionidae).

### Elemental anions and cations in onshore unconventional gas waters

The 10 most studied elements (Supporting Information, Table S1.2) were assessed for statistical significance (*α* ≤ 0.05) between countries (AUS, CAN, CHN, and USA; Figure 6) and between basin type (CBM and shale gas; Figure 5). Significant differences have already been addressed between countries and basins for Cl^−^, Na^+^, Ca^2+^, Mg^2+^, and K^+^ because they are also major ions (see above, *Na* ^*+*^, *Cl* ^*−*^, *and other major ion concentrations in onshore unconventional gas waters*).

The elements Sr, Ba, Fe, Br, and B are addressed below. Between the six unique country pairs (Figure 6) there were only two instances of significantly different pairs, and both were for B between AUS–CAN and CAN–USA. Between basin type CBM and shale gas (Figure 7) Br had a highly significant difference (*α* ≤ 0.001), whereas Sr, Ba, and B had significantly (*α* > 0.001 ≤ 0.05) different concentrations; Fe showed no significance. Across the 10 most studied elements the average concentration was higher in shale gas waters than CBM waters.

Differences between element concentrations in flowback water and produced water with our data set were not evaluated given the low representation of the compiled studies investigating solely flowback water. Despite significant differences by country for some elements in produced water, these differences are fundamentally based on basin type (CBM or shale gas) utilization rates in the countries from our compiled data. Potential differences of these elements by basin (CBM and shale gas) within each country (AUS, CAN, CHN, USA) were not assessed given no shale gas data for AUS and no CBM data for CAN from our literature search and screening.

### Water quality of onshore unconventional gas waters

Water quality data are provided in Supporting Information, Table S1.1, and summary statistics are provided in Supporting Information, Table S4 and presented in Figure 5. For Australian‐based literature (Supporting Information, Table S1.1) TDS data were limited, and when absent an estimated value was determined based on the provided electrical conductivity value and converted by using TDS ≈ electrical conductivity × 0.68 (Hart et al., 1991). The TDS values (Supporting Information, Table S5; Figure 5) in AUS (3900 ± 520 mg/L, *n* = 13) and CHN (16 700 ± 3900 mg/L, *n* = 15) unconventional gas waters were significantly different (*α* ≤ 0.05) from those of CAN (124 000 ± 26 000 mg/L, *n* = 4) and USA (62 700 ± 1300 mg/L, *n* = 28). Although CAN and USA TDS concentrations were not significantly different from each other (α > 0.05), those between CBM (5900 ± 1300 mg/L, *n* = 32) and shale gas (83 200 ± 12 200 mg/L, *n* = 28) basins were highly significant different (α ≤ 0.001).

The pH had highly significant differences (*α* ≤ 0.001) between AUS (8.44 ± 0.096, *n* = 13) and USA (7.09 ± 0.22, *n* = 20) but no significant differences between AUS and CHN, though between CHN (7.67 ± 0.30, *n* = 11) and USA there were no significant differences (*α* > 0.05). No comparisons could be made with CAN because there were no pH values across the limited entries. The pH values between CBM (8.13 ± 0.13, *n* = 30) and shale gas (6.52 ± 0.19, *n* = 13) basins were highly significant (*α* ≤ 0.001). Other WQPs were not further investigated by country or basin because of insufficient data or overlap (electrical conductivity with TDS).

## DISCUSSION

Salinity‐related research from onshore unconventional gas produced water has seen a dramatic increase in interest, with most publications in Supporting Information, Table S1 being published in the last 10 years. The field of acute toxicity for NaCl or SMS to freshwater invertebrates saw a significant increase in publications from the first two decades of the 21st century. At the current time there are no reviews that bridge both research fields on a global scale in a singular review. Folkerts et al. (2020) focus on a North American perspective and the toxicity of flowback produced waters in terms of major ions, trace metals, organics, and whole flowback produced water toxicity. An older review on NaCl toxicity by Hart et al. (1991) focused on salinity sensitivity to Australian freshwater biota. A more recent review of salinity toxicity by Castillo et al. (2018) explores salinity toxicity on a global scale to both vertebrates and invertebrates from freshwater ecosystems and appears to have compiled data from both acute and chronic time points from a wider range of literature, not just peer‐reviewed articles. Our review provides a status quo of the potential salinity toxicity of onshore unconventional gas waters to freshwater invertebrates in receiving environments.

### NaCl toxicity in unconventional gas waters to freshwater invertebrates

There is a clear difference in the two major ions, Na^+^ and Cl^−^, found in CBM and shale gas produced waters (Supporting Information, Table S5, Figure 3). Despite countries also showing significant differences in salinity of associated unconventional gas waters, these differences are mostly attributed to the basin type dominant in each of the countries, where AUS and CHN were either all or mostly CBM, respectively (Supporting Information, Table S1), whereas CAN and USA entries were either all shale gas or dominated by shale gas, respectively, from our compiled literature; these differences between basins are reflected in McGlade et al. (2013) and Silva et al. (2017).

The highly significant differences (*α* ≤ 0.001) in Na^+^ and Cl^−^ ions between CBM and shale gas produced waters can be attributed to depth of well and the geochemistry of these basins where the hydraulic fracturing occurs. Typically, CBM hydraulic fracturing is performed at depths of 300–1400 m below the earth's surface (H. Li et al., 2013; M. Li et al., 2013; Suárez, 2012; Zhang et al., 2005; Zuber, 1998), whereas shale hydraulic fracturing is currently performed at depths between 1500 and 4000 m below the surface (Dong et al., 2018; Jiang et al., 2017; Le, 2018). Coal bed methane basins were formed from plant matter under high precipitation in river basin or swamp environments, followed by accumulation and burial under sediments (Haldar, 2020), whereas many shale gas formations have a marine origin (Folkerts et al., 2020) and consequently can contain high TDS of up to 400 000 mg/L because these waters continue to dissolve surrounding minerals (Benko & Drewes, 2008; Engle et al., 2019) with most of the TDS attributed to Na^+^ and Cl^−^ (Connolly et al., 1990; Folkerts et al., 2019; Kahrilas et al., 2016; Rice, 2003). The shallower depths and relatively more porous nature of CBM basins allow for greater surface freshwater intrusion and replenishment than the deeper and less porous shale gas basins, contributing to increased salinity (Golding et al., 2013).

**Figure 8:**
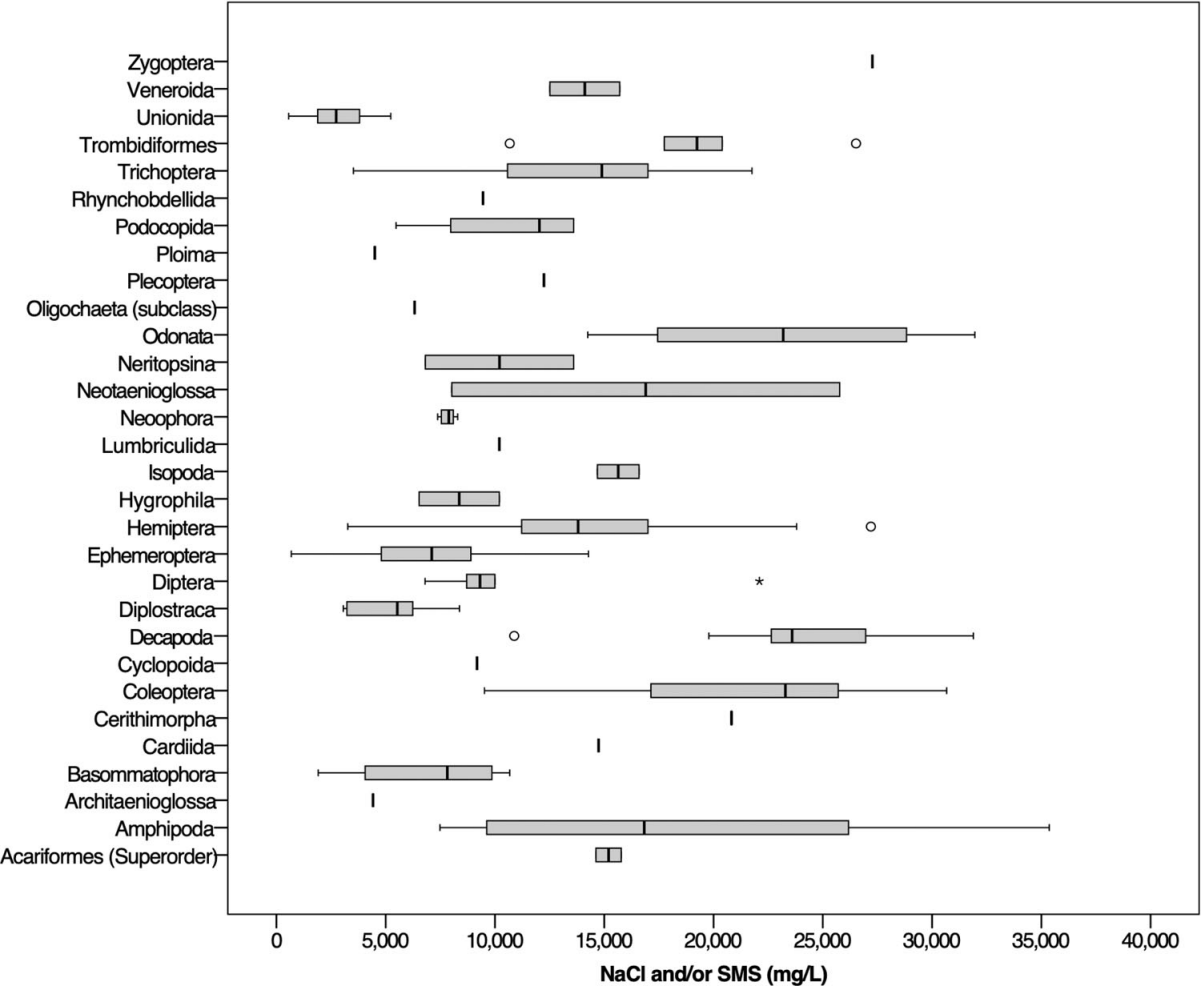
Acute toxicity of NaCl or synthetic marine salts in freshwater invertebrates worldwide, from laboratory‐based tests, categorized by the taxonomic rank of order. Outliers were kept, highlighting the variability of the acute toxicity data among orders.

**Figure 9:**
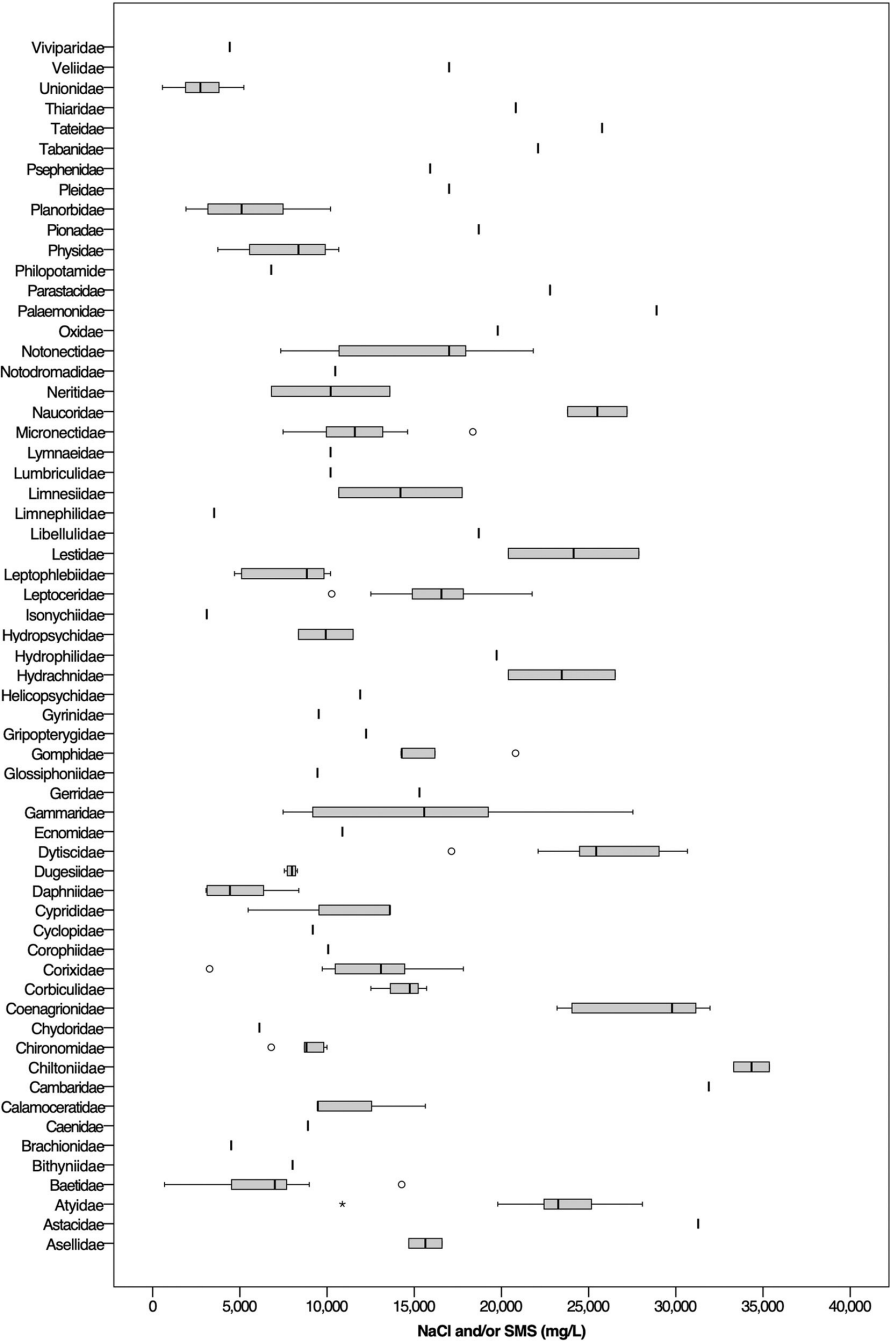
Acute toxicity range of NaCl or synthetic marine salts in freshwater invertebrates worldwide, from laboratory‐based tests, categorized by the taxonomic rank of family. Outliers were kept, highlighting the variability of the acute toxicity data among families.

The average combined Na^+^ and Cl^−^ content for CBM was 4300 ± 1100 mg/L, and that for shale gas was 78 900 ± 10 200 mg/L (Supporting Information, Table S5). These findings are comparable to those of Alley et al. (2011), who concluded that the Cl^−^ content of CBM produced water was <5000 mg/L, whereas that of shale gas produced water was >30 000 mg/L. The Na^+^ and Cl^−^ content alone of untreated CBM produced water would be of low lethal toxicity risk to most freshwater invertebrate orders (Figure 8; Supporting Information, Table S6) and families (Figure 9; Supporting Information, Table S7) if exposed for hours due to spill, short‐duration discharge, or pulse exposure in a laboratory. Some sensitive orders (Unionida, Architaenioglossa, Ploima; Supporting Information, Table S6) and families (Unionidae, Isonychiidae, Limnephilidae, Viviparidae, Brachionidae; Supporting Information, Table S7) could be at an increased risk of experiencing mortality if exposed to the average salinity of untreated CBM produced water for an acute toxicity test duration (48–96 h).

All orders and families of freshwater invertebrates if exposed to the combined Na^+^ and Cl^−^ content of untreated shale gas produced water (78 900 ± 10 200 mg/L) for the duration of an acute toxicity test would almost certainly result in 100% mortality for even the most tolerant taxa (Chiltoniidae) because the average Na^+^ and Cl^−^ content of shale gas produced water is approximately 2.3‐fold the LC50 value for Chiltoniidae (34 300 ± 1000 mg/L). The previous toxicity values reflect a 48–96‐h exposure because they were collected from standardized experimental conditions in the laboratory (ASTM International, 2014; Organisation for Economic Co‐operation and Development, 2004, 2019). A sudden permitted discharge, spill, or leak of saline produced water in an environmental setting could be of a much shorter duration (hours) and would likely be much more realistic in terms of duration in an industry or environmental context where discharges (Veil, 2020) or spills of produced water are common (Alessi et al., 2017; Goss et al., 2015; Patterson et al., 2017). Other factors that may impact the toxicity of a spill should be considered, including the dilution, or flushing, of the spill from the surface water body and the volume of the discharge, spill, or leak relative to the volume of the surface water body.

There is sparse literature that has assessed pulse toxicity of NaCl to freshwater organisms (Diamond et al., 2005; Fleetwood, 2017). In the study by Diamond et al. (2005), 6‐day‐old larval fathead minnows were pulsed once at 12 g/L for 3 h with NaCl within the first 24 h of a 7‐day chronic test, resulting in a 62% survival rate after 7 d. This endpoint also is closely comparable to the 8‐g/L pulse for 24 h with NaCl and a 59.3% survival after 7 days. Fleetwood (2017) investigated 96‐h acute toxicity of NaCl using pulse only and pulse with a rest/recovery period exposures to freshwater invertebrates belonging to Heptageniidae—*Maccaffertium* and *Epeorus*. Several treatment conditions were conducted, though of interest were the 10 000 μS cm^–1^ pulse only and the 10 000 μS cm^–1^ (≈6800 mg/L NaCl) with 900 μS cm^–1^ (≈612 mg/L NaCl) rest experiments. *Maccaffertium* and *Epeorus* with a 1‐h pulse of NaCl had 96‐h survival of 100% and 87.5% for the pulse‐only and rest treatments, respectively; with a 6‐h pulse of NaCl survival had dropped to 70% and 52.5% for pulse only and rest treatments, respectively, indicating that even after the exposure at higher salinity had ceased, mortality continued during the rest/recovery period.

If a spill resulting in a short exposure of CBM produced water were to occur based on average NaCl content found in our literature search (4300 ± 1100 mg/L), it would appear from the limited pulse toxicity literature for NaCl that freshwater invertebrates and fish would be able to tolerate a spill of CBM produced water, particularly if it was continually flushed or renewed with flowing water in a stream or river environment. A spill of shale gas produced water, though based on the combined Na^+^ and Cl^−^ content (78 900 ± 10 200 mg/L) found in our literature search would be quite detrimental to the health of surface water bodies, particularly with minimal flow or a low volume of water. It should be highlighted that the toxicity values above are from single‐species laboratory toxicity tests and might be overestimating the actual toxicity endpoints that could be expected in an ecosystem or a simulated environment like a mesocosm (Odum, 1984), if produced waters were to be discharged, leaked, or spilled into a surface water body (see section *Impact of unconventional gas secondary salinization in surface waters*).

The CBM and shale gas basin geochemistry are complex and unique to each well. Concerns with salinity toxicity of produced water should ideally be assessed on a case‐by‐case basis. With extra caution for shale gas produced water given that these waters are more likely to have highly elevated Na^+^ and Cl^−^ and other major ions that could be detrimental or even lethal if a discharge, spill, or leak to surface water ecosystems was to occur. Though high Na^+^ and Cl^−^ concentrations of raw untreated produced water are usually the major toxicity concern, other components such as other major ions (Ca^2+^, K^+^, Mg^2+^, ${\text{CO}}_{3}^{2-}$, HCO_3_^−^, and ${\text{SO}}_{4}^{2-}$), dissolved nonessential or toxic metals, and organics may also be of concern. There is also limited evidence that despite SMS being mostly composed of Na^+^ and Cl^−^, SMS has a lower toxicity compared to NaCl (Kefford, Palmer, et al., 2004).

### Influences of other major ions to freshwater invertebrate toxicity

Though Na^+^ and Cl^−^ are typically the two major ionic components in unconventional gas waters, other major geochemical ions such as Ca^2+^, K^+^, Mg^2+^, ${\text{CO}}_{3}^{2-}$, HCO_3_^−^, and ${\text{SO}}_{4}^{2-}$(Erickson et al., 2017; Mount et al., 2019) influence the toxicity Na^+^ and Cl^−^. Mount et al. (1997) stated that in their multiple logistic regression model for the three organisms they tested (*Ceriodaphnia dubia*, *Daphnia magna*, and *Pimephales promelas*), in general, relative ion toxicity was K^+^ > HCO_3_^−^ ≈ Mg^2+^ > Cl^−^ > ${\text{SO}}_{4}^{2-}$.

We stress that the following examples in toxicity interactions between major ions and freshwater invertebrates are only a small representation of the diversity and that other biota may respond differently from the examples provided. We also stress that the interactions between all major ion pairs have not been discussed in the present review for the sake of conciseness and are further detailed in the literature (Erickson et al., 2017, 2022; Mount et al., 2016, 2019; Soucek & Kennedy, 2005; Soucek et al., 2011).

Potassium (K^+^) and its influence on Na^+^ toxicity was investigated in Mount et al. (2016) using *C. dubia*. They found that at 1.62 and 300 mg Na^+^/L the respective 48‐h K^+^ LC50 values were 80.2 and 394.3 mg/L. They concluded that increasing Na^+^ has an ameliorative effect on K^+^‐containing salts. The elevated toxicity of K^+^ with low Na^+^ is unlikely to occur in unconventional gas waters where Na^+^ greatly exceeds K^+^ concentrations, particularly in shale gas produced water (Figure 3; Supporting Information, Table S5).

The water hardness Ca^2+^ and Mg^2+^ ions can influence Cl^−^ toxicity. Soucek et al. (2011) assessed the effects of varying water hardness (Ca^2+^ and Mg^2+^) and its impacts on 48‐h Cl^−^ acute toxicity in *C. dubia*. At 50 and 800 mg/L hardness the respective 48‐h Cl^−^ LC50 values were 860 and 1840 mg/L; thus, water hardness has an ameliorative effect on acute Cl^−^ toxicity in *C. dubia*. Soucek et al. (2011) suggest that the most likely explanation for this phenomenon is that cellular junctions are tightened by increased concentrations of Ca^2+^ and, to a lesser extent, Mg^2+^ (Potts & Fleming, 1970), thus reducing passive diffusion of Cl^−^ into cells and the energy required for ionoregulation. Soucek et al. (2011) also investigated water hardness on 96‐h Cl^−^ acute toxicity to three other organisms (*Sphaerium simile*, *Gyraulus parvus*, *Tubifex tubifex*) for the effects of water hardness at hardness ≈50 and ≈200 mg/L for each organism. Both *S. simile* and *T. tubifex* had marked decreases in 96‐h acute toxicity responses with increased hardness from ≈50 to ≈200 mg/L, whereas *G. parvus* had very similar 96‐h acute responses between the two water hardness tiers.

Based on the evidence in Soucek et al. (2011), the elevated Ca (6800 mg/L) and Mg (800 mg/L) in the much harder shale gas waters compared to CBM waters, Ca (96 mg/L) and Mg (31.2 mg/L; Figure 3; Supporting Information, Table S5), is likely to reduce the toxicity of the high‐Cl^−^ ions present in shale gas produced water (Figure 3), though the concentrations of Ca and Mg in shale gas waters could have potential lethality to model organisms such as *Daphnia* spp. For Ca in Dowden & Bennett (1965), *D. magna* had a 48‐h LC50 for CaCl_2_ at 3000 and 760 mg/L or as Ca content of 1080 and 270 mg/L, respectively (two different culture media were used). In Baudouin & Scoppa (1974), *Daphnia hyalina* had a 48‐h LC50 for Ca (as CaCl_2_ × 2H_2_O) at 3000 mg/L. In Mount et al. (1997), *C. dubia* had a 48‐h LC50 for CaCl_2_ at 1830 mg/L or as Ca at 660 mg/L, whereas *D. magna* had a 48‐h LC50 for CaCl_2_ at 2770 mg/L or as Ca at 1000 mg/L. For Mg (as MgCl_2_) in Baudouin & Scoppa (1974), *D. hyalina* had a 48‐h LC50 for Mg (as MgCl_2_ × 6H_2_O) at 32 mg/L. In Mount et al. (1997), *C. dubia* had a 48‐h MgCl_2_ LC50 of 880 mg/L or as Mg at 225 mg/L, whereas *D. magna* had a 48‐h MgCl_2_ LC50 of 1330 mg/L or as Mg at 340 mg/L, and in Dowden & Bennett (1965) *D. magna* had a 48‐h MgCl_2_ LC50 or as Mg at 945 mg/L.

The interaction of water hardness and ratios of Ca:Mg and their influence on the toxicity of ${\text{SO}}_{4}^{2-}$ in *D. magna* and *Hyalella azteca* have been investigated (Davies & Hall, 2007). They found that an increase in water hardness from 25 to 250 mg/L significantly increased the 96‐h ${\text{SO}}_{4}^{2-}$LC50 of *H. azteca* from 570 to 5300 mg/L, respectively. In *D. magna* with a 25 to 100 mg/L increase in hardness the 48‐h ${\text{SO}}_{4}^{2-}$ LC50 increased from 1200 to 3200 mg/L, respectively. Modification of the Ca:Mg ratios to 0.7 and 7.0 while maintaining a constant overall hardness of 100 mg/L also significantly increased the 96‐h ${\text{SO}}_{4}^{2-}$ LC50 values in *H. azteca* from 2100 to 2700 mg/L. Using the same Ca:Mg ratios as above at an overall hardness of 25 mg/L, in *D. magna* the 48‐h ${\text{SO}}_{4}^{2-}$ LC50 values increased from 1200 to 2000 mg/L; and at 100 mg/L constant hardness with the same ratios again, the 48‐h ${\text{SO}}_{4}^{2-}$ LC50 increased from 3200 to 4400 mg/L. Davies & Hall (2007) state that NaCl and KCl toxicity to *D. magna* was not significantly different in waters with different Ca:Mg ratios. From the evidence provided in the Davies & Hall (2007) study it would be reasonable to expect that in shale gas produced waters the average concentrations of Ca (6800 mg/L) and Mg (800 mg/L; Figure 3; Supporting Information, Table S5) at an approximate ratio of 8.5 Ca:1 Mg would reduce ${\text{SO}}_{4}^{2-}$ toxicity in these waters.

The ratios of Ca:Mg have also been shown to influence HCO_3_^−^ toxicity in *C. dubia* as in Mount et al. (2016) using NaHCO_3_. A low and a high Ca:Mg treatment resulted in 48‐h NaHCO_3_ LC50s of 1540 and 2000 mg/L, respectively, or 1120 and 1450 on an HCO_3_^−^ basis, respectively. Both CBM and shale gas waters have a high Ca:Mg ratio of approximately 3:1 and 8.5:1, respectively (Supporting Information, Table S5). Thus, the higher Ca:Mg ratios seen in both CBM and shale gas show that it would be reasonable to expect that HCO_3_^−^ toxicity would be reduced.

The influence of ${\text{SO}}_{4}^{2-}$ on Cl^−^ toxicity was investigated in Soucek et al. (2011). They found that Cl^−^ toxicity slightly increased with increasing ${\text{SO}}_{4}^{2-}$ concentrations in *C. dubia*. At 26 and 712 mg/L ${\text{SO}}_{4}^{2-}$ the respective 48‐h Cl^−^ LC50s were 1360 and 1200 mg/L. The concentrations of ${\text{SO}}_{4}^{2-}$ tested in Soucek et al. (2011) were comparable to mean values in CBM (120 mg/L) and shale gas (210 mg/L) waters (Figure 3; Supporting Information, Table S5).

The study by Hills et al. (2019) compared the acute toxicity of the alkalinity ion (HCO_3_^−^) to an SMS in eight different organisms, which were approximately 2–35‐fold more sensitive to NaHCO_3_ compared with the SMS. For six of the eight organisms the 96‐h LC50 NaHCO_3_ concentrations were 800–4300 mg/L or on an HCO_3_^−^ basis 580–3100 mg/L. The average HCO_3_^−^ concentration found in CBM waters (1700 mg/L; Supporting Information, Table S5) would likely influence the toxicity of these waters, particularly to a NaHCO_3_‐sensitive species like *Paratya australiensis* (Hills et al., 2019; Vera et al., 2014). The average HCO_3_^−^ content in shale gas waters (363 mg/L; Supporting Information, Table S5) would be of less concern, though shale gas waters generally have far greater concentrations of most other major ions (Figure 3; Supporting Information, Table S5) and other elements (Sr, Ba, Br, B; Figure 7). Hoke et al. (1992) used X‐ray dispersive microanalysis to study intracellular Cl^−^ concentrations in *D. magna* that were exposed to elevated HCO_3_^−^ concentrations and found that intracellular Cl^−^ concentrations were depleted. They suggested that a Cl^−^/HCO_3_^−^ exchanger across the gill membranes was possibly being inhibited by the exposure to elevated HCO_3_^−^ concentrations, resulting in *D. magna* being unable to maintain a hemolymph and an intracellular acid–base balance (Hoke et al., 1992).

The alkalinity ion ${\text{CO}}_{3}^{2-}$ was investigated in Mount et al. (2016) as CaCO_3_ at a low (10 mg/L) and a high (90 mg/L) concentration or on a ${\text{CO}}_{3}^{2-}$ basis of 6 and 53.9 mg/L and its influence on 48‐h *C. dubia* acute toxicity against major ions using the salts KCl, NaCl, MgCl_2_, Na_2_SO_4_, and MgSO_4_. The higher CaCO_3_ concentrations generally increased the toxicity of these salts by approximately 10%–20%, though it was slightly reduced in the NaCl combination, where at 6 and 53.9 mg/L ${\text{CO}}_{3}^{2-}$ (as CaCO_3_) the 48‐h LC50 was 1860 and 1970 mg/L NaCl, respectively. The high 53.9 ${\text{CO}}_{3}^{2-}$concentration investigated in Mount et al. (2016) represents a value similar to those found in average CBM (79.4 mg/L) and shale gas (90.0 mg/L) water samples (Figure 3; Supporting Information, Table S5).

### Impact of unconventional gas secondary salinization in surface waters

Secondary salinization is a growing ecological issue. Climate change and many industrial processes (particularly agriculture) are contributing to secondary salinization of surface waters (Cañedo‐Argüelles et al., 2013). A relevant example is the permitted discharges, spills, or leaks of chemically complex unconventional gas produced water (Elsner & Hoelzer, 2016; NICNAS, 2017; Waxman et al., 2011) to surface waters (Olmstead et al., 2013; Vengosh et al., 2014; Warner et al., 2013). Unlike the previously discussed NaCl and/or SMS toxicity from laboratory‐based single–test organism experiments from the literature, surface water ecosystems are vastly more complex with many direct and indirect effects or factors that can influence chemical toxicity, as detailed in the review by Cañedo‐Argüelles et al. (2013). There is a paucity of research in mesocosm (Odum, 1984) studies that assess the toxicity of salinity/major ions as pulses, repeated pulses (Cañedo‐Argüelles et al., 2014), or short‐term exposures (Cañedo‐Argüelles et al., 2012), which would be more relevant in the scenario of permitted discharge, leak, or spills of produced waters into surface waters.

Cañedo‐Argüelles et al. (2014) found that repeated NaCl pulse exposure to algae (diatoms) and invertebrates using a stream mesocosm led to a significant (*α* < 0.001) decline in invertebrate density (total number of invertebrates) across the low (5 mS cm^–1^, ≈3400 mg/L), medium (10 mS cm^–1^, ≈6800 mg/L), and high (15 mS cm^–1^, ≈10 200 mg/L) treatments relative to the control (0.64 mS cm^–1^, ≈435 mg/L) at day 7. By day 16, there was noticeable recovery, though this was less pronounced in the high treatment, indicating possible and greater adaptation to less severe NaCl changes. Across both diatoms and invertebrates the taxa richness had not significantly changed throughout the experiment. Community composition (abundance of the different taxa) was most different at day 11 in the treatments compared to the control. Cañedo‐Argüelles et al. (2014) highlight that most of the invertebrates were able to resist the first set of salt pulses, but then the population began to perish after two more spikes, probably because of reduced fitness caused by pulsing (Ashauer et al., 2007).

Concentrations of NaCl in Cañedo‐Argüelles et al. (2014) are relevant to those found in CBM and shale gas produced waters, though a discharge, spill, or leak of produced waters into surface waters will quickly be diluted by the relatively larger‐volume or high–flow rate surface waters, reducing the toxicity. The much higher‐salinity shale gas produced water (Figure 3) would produce a more pronounced spatial salinity gradient (Annevelink et al., 2016; Olmstead et al., 2013) compared with the less saline CBM produced waters (Figure 3), though it would be more toxic closer to the discharge or spill origin (Olmstead et al., 2013). Though not ideal, if discharging shale gas produced water is permitted (NPDES) it should only occur in large, fast‐flowing surface waters that can rapidly dilute the high salinity. However, there are still many other toxic components (Neff et al., 2011) in these waters, some at relatively high levels such as the metals Ba (810 mg/L) and Sr (1200 mg/L; Figure 7) that readily precipitate with sufficient ${\text{SO}}_{4}^{2-}$ concentrations and thus may settle to the sediment (Ferrar et al., 2013) and may chronically impact sediment‐dwelling biota.

To the authors' knowledge, based on a literature search, there has not been a mesocosm‐based approach to investigate the whole‐effluent toxicity of an onshore unconventional gas water sample to freshwater invertebrates. If this mesocosm study involved pulse(s) of raw/untreated produced water, it would better simulate the environmentally realistic release of a discharge, spill, or leak of these waters and how they might impact a complex freshwater surface water ecosystem. Compared with testing produced waters with a single species at a time in a laboratory (Blewett, Delompré, et al., 2017; Blewett, Weinrauch, et al., 2017; Delompré et al., 2019; Folkerts et al., 2017a, 2017b, 2019; Golding et al., 2022; He et al., 2017).

### Geographical and basin type variation of elements and ions

The 10 most studied elements (Supporting Information, Table S1.2) compared between CBM and shale gas basins (Figure 7) provide insight that basin type is a major factor contributing to the different elemental profiles between them. Despite observing significant differences between countries (Figure 6), the differences by countries can be attributed to the basin type that was dominant in that country (from the obtained literature): CBM for AUS and CHN, shale gas for CAN and USA (Supporting Information, Table S1; see *Results*).

The extraction of natural gas from CBM and shale gas occurs at different depths below the earth's surface as detailed earlier (see *NaCl toxicity in unconventional gas waters to freshwater invertebrates*). It is not unreasonable to expect differences in elements between these two basin types that are influenced by depth and the resulting geochemical differences. Silva et al. (2017) also found maximum values of various elements to be higher in shale gas produced waters compared with CBM produced water.

There is an array of other factors that may also impact the dissolved element concentrations. These can include leaching, adsorption, redox, and water mixing (Tong et al., 2019); mineral composition (Ahamed et al., 2019); natural water content of the formation (Jamshidi & Jessen, 2012; Moore, 2012); reservoir characteristics such as porosity and permeability (Moore, 2012); time since initiation of pumping, well spacing, and density (Kondash et al., 2017); and methanogenic organisms that are able reduce heavy metals (Colosimo et al., 2016) and WQPs (e.g., pH and temperature).

The effect of acidic pH and its influence on increased dissolution of metals from sediments and minerals, and the resulting increased solubility of these metals in solution, is a well‐known phenomenon (Calmano et al., 1993; Chuan et al., 1996; Gambrell, 1994; Li et al., 2013). From our findings from the literature the mean pH of shale gas and CBM waters was 6.52 and 8.13, respectively. Chuan et al. (1996) assessed the solubility of Pb, Cd, and Zn with changing pH and found that these metals were sparingly soluble at pH 8 and that solubility increased with greater acidification at pH 5 and then again at pH 3. Li et al. (2013) also observed that heavy metals were released much more readily from sediments at lower pH 4–7 compared to pH 8–10. The differences in pH between CBM and shale produced waters could explain the observed differences in concentrations in produced water for the more commonly studied metals between these basins (Figure 7) and the alkalinity ion of HCO_3_^−^ (Figure 3; Supporting Information, Table S5). Because bicarbonate in waters in the environment is derived from natural sources, it typically has higher HCO_3_^−^ concentrations, peaking at approximately pH 8 (Ghobadi et al., 2016), which is similar to the pH 8.13 in CBM waters, and becomes reduced as pH decreases (or increases) as in shale gas waters with a mean pH of 6.52.

### Toxicity of untreated onshore unconventional gas water samples

Despite salinity typically being the most toxic component of produced water (Folkerts et al., 2019), in particular for shale gas produced water, it is important to not disregard the other chemicals within produced water that can originate from their use in hydraulic fracturing fluids or a geogenic (groundwater) source (Elsner & Hoelzer, 2016; NICNAS, 2017; Waxman et al., 2011). Other factors impacting toxicity of these waters include when the flow backwater or produced water is sampled relative to the initial hydraulic fracturing of a well, resulting in temporal changes of chemical concentrations (Barbot et al., 2013; Cluff et al., 2014; Folkerts et al., 2019). Parameters such as pH and dissolved oxygen (Figure 6; Supporting Information, Table S4) are known factors that can result in physiological stress of organisms if outside tolerable levels to a specific organism and can interact synergistically with other classes of chemicals (Holmstrup et al., 2010). The high salinity concentrations in shale gas produced water can also interact synergistically with toxic dissolved ions (Hall & Anderson, 1995; McLusky et al., 1986) that are frequently found in elevated concentrations (Figure 5) in unconventional gas waters (Alley et al., 2011).

There has been a recent increase in literature that has assessed the toxicity of raw or untreated samples of whole‐effluent water samples of flowback water or produced waters from unconventional gas (Blewett, Delompré, et al., 2017; Blewett, Weinrauch, et al., 2017; Delompré et al., 2019; Folkerts et al., 2017a, 2017b, 2019; Golding et al., 2022; He et al., 2017). Folkerts et al. (2019) investigated the acute toxicity of flowback produced water collected at three different time points (1.33, 72, and 228 h) from the same well and a salinity control in the organisms *D. magna*, *Lumbriculus variegatus*, *Danio rerio* embryos, and *Oncorhynchus mykiss* (embryos and juveniles). Only referring to their LC50 data for conciseness, it was observed that the toxicity of their three temporal flowback produced water samples was highly different for each of the organisms, particularly when comparing *O. mykiss* data to the other three organisms, likely due to major physiological differences between these organisms. The toxicity of the three different time points of flowback produced water sampled varied for each organism, though generally toxicity was higher in the 1.33‐h, compared to the 72‐ and 228‐h, samples, though this was not the case for *D. rerio* embryos, likely due to the much more elevated concentrations of certain organics in the 1.33‐h sample than the 72‐ and 228‐h water samples (Folkerts et al., 2019). Most of the organisms were able to tolerate the salinity control better than the three temporal samples of flowback produced water, with exception of *D. rerio* embryos and *L. variegatus*. *O. mykiss* embryos and juveniles were approximately twice as sensitive to the 1.33‐h flowback produced water sample (96‐h LC50 of 8.82% and 7.14% v/v, respectively) compared to the salinity control (96‐h LC50 of 16.87% and 14.24% v/v, respectively). Folkerts et al. (2019) showed that even though salinity is the major toxicity concern of unconventional gas produced water. Other groups of chemicals (organics and inorganics) should not be completely disregarded because they may alter the toxicity of the whole flowback produced water sample and make some organisms more sensitive to a given water sample. Golding et al. (2022) found that a 1 in 1140 dilution (0.088%) of a shale gas produced water sample (TDS = 238 000 mg/L, Na^+^ and Cl^−^ content = 157 300 mg/L) was required to prevent chronic adverse effects in 95% of freshwater biota.

### Salinity toxicity of treated versus untreated produced waters

Multiple methods exist for treating onshore unconventional gas produced water to reduce the high salinity content, metals, organics, and microorganisms (Abousnina et al., 2015; Al‐Ghouti et al., 2019; Chang et al., 2019; Nasiri et al., 2017; Silva et al., 2017), the focus usually being salinity because it is frequently the most problematic component. High salinity restricts the ability to reuse, recycle, or repurpose produced water. Reverse osmosis is a highly effective and established method to sharply reduce dissolved or suspended components in water. It can see removal efficiencies of ions of >98% and of TSS and oil and grease of >90% from produced water (Al‐Ghouti et al., 2019; Alzahrani et al., 2013; Mondal & Wickramasinghe, 2008).

After reverse osmosis treatment of shale gas produced water and assuming the 98% removal efficiency threshold of dissolved ions, the mean Na^+^ and Cl^−^ content would be <1557 mg/L from raw samples of 78 900 ± 10 200 mg/L (Figure 3; Supporting Information, Table S5). The reverse osmosis‐treated shale gas produced water would be regarded as slightly saline water (Swenson & Baldwin, 1965). For reverse osmosis‐treated CBM produced water the Na^+^ and Cl^−^ content would be <87 mg/L from raw samples of 4300 ± 1100 mg/L (Figure 3; Supporting Information, Table S5), which would be regarded as freshwater (Swenson & Baldwin, 1965). This would result in both the more saline shale gas produced water and the less saline CBM produced water being at concentrations below the most NaCl and/or SMS‐sensitive freshwater invertebrate order (Figure 9; Supporting Information, Table S6) and family (Figure 7; Supporting Information, Table S7) taxa.

Though reverse osmosis can solve the highly saline shale gas produced water, the implementation of reverse osmosis treatment for the unconventional gas industry faces multiple challenges. Pretreatment of unconventional gas produced water is required before reverse osmosis treatment to reduce excessive wear, fouling, and clogging of the reverse osmosis filters (Malaeb & Ayoub, 2011; Ozgun et al., 2013). The large volumes of flowback and produced water produced were estimated to be 1.7 to 14.3 × 10^6^ L per well (Kondash et al., 2017). Other challenges include the financial cost and time investment to construct reverse osmosis facilities and then the operating costs of these facilities, such as electricity and membrane replacements to run these systems (Malaeb & Ayoub, 2011; Meng et al., 2016); managing and disposal of the highly concentrated sludges (Malaeb & Ayoub, 2011); and the transportation of large volumes of untreated or partially pretreated produced water to these facilities and then the transport required for the disposal of brine concentrate post–reverse osmosis treatment (Meng et al., 2016). Silva et al. (2017) have summarized the estimated costs for transportation, storage, and disposal of produced water.

## CONCLUSION

We found that there are generally vast differences in the two major salinity (Na^+^ and Cl^−^) ions, other major ions (Ca, K, Mg, ${\text{CO}}_{3}^{2-}$, HCO_3_^−^, and ${\text{SO}}_{4}^{2-}$), and various other elements between shale gas and CBM produced waters, with shale gas produced waters usually having more elevated levels compared with CBM produced waters. The origin of these waters, the surrounding geochemistry of coal beds and shales, influence the pH of these waters and can affect the concentrations of various ions. These findings can impact how produced waters from CBM or shale gas basins could be treated or managed to lower the toxicity of these waters for secondary or beneficial reuse. It was found that most freshwater invertebrates would likely be able to tolerate and survive an exposure based on the Na^+^ and Cl^−^ from a discharge, spill, or leak of CBM produced waters and even for the duration of laboratory acute toxicity exposures of 48–96 h, though it must be highlighted that other major ions influence the toxicity of Na^+^ and Cl^−^ and other major ions. Organisms that are sensitive to bicarbonate might be at particular risk to the higher bicarbonate content in CBM produced waters. Meanwhile, even a short‐duration spill, leak, or discharge of untreated shale gas produced water is likely to have severe impacts on all freshwater invertebrate life and other freshwater biota in general, particularly if the shale gas waters remain relatively concentrated in a surface water body because of it having a relatively low flow rate and volume. Golding et al. (2022) showed that they needed a 1 in 1140 dilution (0.088%) for a “safe” dilution to prevent chronic adverse effects in 95% of freshwater biota. Onshore unconventional gas produced waters contain a complex mix of chemicals and other toxic components such as inorganics (metals) and organics that will also influence the overall toxicity of these waters and that should also be considered.

Despite the published data for salinity in onshore unconventional gas produced waters being plentiful, the reporting alongside of other WQPs and assessment of metals are highly variable, making it a challenge to perform meaningful statistics with sufficient statistical power. Future studies should be encouraged to report a sweep of WQPs and metals, guided by those most frequently reported from our review. There are also sparse measurements of organics when the salinity of produced water is a focus of the literature, and increased reporting of common and major organics in these waters should be investigated more regularly. We would also like to highlight for future studies investigating taxonomic differences in toxicity that they ensure that taxonomic classifications are correct and up to date at the time of publishing because one piece of literature had reported class classifications under a heading titled *order*. An interesting avenue of further research could be to conduct a mesocosm‐based experiment using a pulsed exposure of a whole untreated/raw unconventional gas produced water. This would more completely simulate the release of these waters as a discharge, spill, or leak and how they might impact complex freshwater surface ecosystems.

## Supporting Information

The Supporting Information is available on the Wiley Online Library at https:/10.1002/etc.5492.

## Conflict of Interest

The authors declare that they have no known competing financial interests or personal relationships that could have appeared to influence the work reported in the present study.

## Author Contributions Statement

**Daniel J. Willems**: Conceptualization; Data curation; Formal analysis; Investigation; Methodology; Writing—original draft; Writing—review & editing. **Anu Kumar**: Conceptualization; Supervision; Writing—review & editing. **Dayanthi Nugegoda**: Conceptualization; Supervision; Writing—review & editing.

## Supporting information

This article includes online‐only Supporting Information.

Supporting File.

## Data Availability

Data are available from the corresponding author (daniel.willems@student.rmit.edu.au) and from https://doi.org/10.17632/bt76wh8v5d.1.
